# Composition and Morphological Characteristics of Extracellular Polymeric Substances of Different Tolerant Bacteria Under Perfluorobutanesulfonic Acid (PFBS) Stress

**DOI:** 10.3390/toxics12110797

**Published:** 2024-10-31

**Authors:** Rui Tang, Lina Sun, Guo Yu, Jiayao Xu, Qing Luo, Xiaoxu Wang, Luge Rong

**Affiliations:** 1 Key Laboratory of Regional Environment and Eco-Remediation, Ministry of Education, Shenyang University, No. 21 Wanghua South Street, Dadong District, Shenyang 110044, China; 2 School of Biomedical and Chemical Engineering, Liaoning Institute of Science and Technology, Benxi 117004, China

**Keywords:** perfluorobutanesulfonic acid, extracellular polymeric substances, protein, polysaccharides, bacterial strain, biosorption

## Abstract

This investigation studies the properties and composition of extracellular polymeric substances (EPS) of the four tolerant bacterial strains [NH (*Cellulosimicrobium cellulans)*, TH, YH, and HE (*Pseudomonas aeruginosa*)] under perfluorobutanesulfonic acid (PFBS) stress. The strains were acquired from athickened sludge in a fluorine chemical park. Each strain’s EPS were isolated by heating and centrifugation, and their growth, metabolic activity, and EPS alteration research pre- and post-stress were assessed and compared. The strain type was identified by morphological observation and 16S rDNA gene sequence analysis. Under PFBS (100 μg·L^−1^) stress, the four tolerant strains NH, TH, YH, and HE showed 38.10%, 29.26%, 35.92%, and 30.48% removal of PFBS on day 4, respectively, and the strain’s EPS had a substantial impact on main component protein (PR) and polysaccharide (PS) contents. The NH microorganism’s ability to metabolize organic matter was markedly stronger; it had a higher quantity, and its impact on main EPS content was greater than the other three tolerant strains. The three-dimensional excitation–emission matrix results showed marked alterations in tryptophan and aromatic protein peaks in the tolerant strain’s EPS. Furthermore, the FTIR analysis showed that the intensity of the functional groups in the proteins (-OH, C=O, -NH, and -CN) and the polysaccharides (-OH, C-O-C, and C-O) changed significantly. This investigation indicated that the proteins and polysaccharides of the tolerant strain’s EPS could provide more binding sites for PFBS adsorption, where the NH strain had the best biosorption capacity. This research provides a theoretical basis for elucidating efficient biosorbents.

## 1. Introduction

Perfluorinated compounds (PFCs) are synthetic compounds in which every carbon (C) attached hydrogen atom is replaced by fluorine atoms, giving them very high chemical stability even under severe conditions such as high temperature and pressure [1]. Perfluorinated sulfonic acids (PFSAs) are perfluorinated compounds that mainly comprise perfluorooctane sulfonic acid (PFOS), perfluorooctane sulfonic acid (PFHxS), and perfluorobutane sulfonic acid (PFBS). Long-chain PFSAs (with >6 perfluoroalkyl carbons) have been widely utilized in consumer goods production, such as in textiles, non-stick cookware, foam fire extinguishing agents, food packaging, and carpet protectors, for a long time [2,3,4,5]. PFOS and PFBS are the main PFSAs present in the environment [6,7]. In 2009, the Stockholm Convention declared PFOS (the most widely used PFSA) hazardous due to its environmental persistence, high toxicity, bioconcentration, and widespread pollution, and its production and use were restricted [8]. Simultaneously, PFBS, a short C-chain PFSA, was being used as a substitute for PFOS [9], resulting in frequent PFBS detection in surface water, landfill leachate [10], and soil [4]. It has been reported in the literature that PFBS can accumulate in the tissues of several freshwater and marine fishes [11]. The refractory nature of PFBS allows it to enter organisms through bioconcentration and cause direct or indirect damage and toxic effects, especially to humans, including in the nerves, reproductive development system, and other organs [12]. Since the solubility of PFBS (42 g·L^−1^) is much greater than PFOS (591 mg·L^−1^) [13], its high environmental persistence and hydrophilicity make it a higher global pollutant than PFOS. PFBS and its salts were officially declared candidates for the substance of very high concern on 16 January 2020. Therefore, an investigation of feasible methods for efficient PFBS removal from the environment is urgently required.

At present, only a few reports on the remediation of short C-chains PFBS exist. However, since it has a PFOS-like physical structure and elemental composition, the existing processes for PFOS removal can be considered for optimizing the reaction conditions and parameters to achieve PFBS removal. Physical methods such as adsorption [14] and membrane separation [15] can separate PFOS from the environment but cannot achieve complete removal. Chemical methods such as UV reduction [16] can effectively degrade PFCs at high concentrations in water. Still, the degradation efficiency of this method is low for pollutants in low concentrations. Although microbial degradation is not the main PFBS remediation process [17], it is considered for PFBS removal because of its high efficiency, low secondary pollution production, low cost, and low PFBS concentration in most actual environments. Therefore, it is essential to research tolerant strains of short-chain PFCs and microbial remediation.

Extracellular polymeric substances (EPS) are organic polymeric compounds released by microorganisms outside the body or cell to create a protective layer against stressful external environmental conditions [18,19]. It is also an energy and C reserve [20,21]. EPS form a buffer layer outside the cell to furnish relatively stable conditions for microbial growth, which is a self-protection mechanism against adverse external influences (e.g., heavy metals, and toxic organic compounds). In combination with the EPS functional groups, including hydroxyl (-OH), carboxyl (-COOH), and sulfhydryl (-SH), the organic matter decreases the mobility and toxicity of pollutants [22]. Weathers et al. [23] found that PFCs induce microorganisms to secrete more EPS, which protects them from toxic substances and ensures their growth and reproduction. Yan et al. [24] found that in wastewater treatment plant sludge, the amide and aromatic groups on EPS protein structures interact with -COOH heads and C-F chains of perfluorooctanoic acid (PFOA) via electrostatic attraction and hydrophobic interaction, respectively. The EPS proteins could furnish more binding sites for PFOA adsorption. At present, related research primarily focuses on the effects of long-chain PFCs on EPS, while investigations on the effects of short-chain PFCs on EPS are relatively rare.

Therefore, in this investigation, four different PFBS-tolerant strains were selected, EPS were extracted by heating method, PFBS was selected as the target pollutant, and PFBS adsorption by the tolerant strains’ EPS was used as the entry point to explore the characteristic effects of PFBS stress on different tolerant EPS strains. This was performed by identifying the main EPS content and tolerant strains, characterizing the tolerant strain’s EPS, determining PFBS residues, and demonstrating the feasibility of microbial remediation of PFC’s environmental contamination.

## 2. Materials and Methods

### 2.1. Sample Acquisition

The samples were taken from the surrounding area of the fluorine chemical park wastewater treatment plant in Fuxin, Liaoning Province, China, in October 2020. Sludge and soil were sampled using a metal spatula in 2 L polypropylene bottles and placed in an ice box before storage at 4 °C in the laboratory for subsequent experiments.

### 2.2. Medium and Chemicals

The enrichment culture medium contained NH_4_NO_3_ (5 g/L), K_2_HPO_4_ (1 g/L), KH_2_PO_4_ (1 g/L), MgSO_4_·7H_2_O (0.5 g/L), NaCl (2 g/L), CaCl_2_·2H_2_O (0.05 g/L), yeast extract (1 g/L), and different PFASs concentrations (200, 300, 400, and 500 mg/L), pH (7.0).

The inorganic salt culture media comprised NH_4_NO_3_ (5 g/L), KH_2_PO_4_ (1 g/L), KH_2_PO_4_ (1 g/L), MgSO_4_·7H_2_O (0.5 g/L), NaCl (2 g/L), CaCl_2_·2H_2_O (0.05 g/L), pH (7.0), and different PFASs concentrations as the only C source.

The modified LB media comprised peptone (10 g/L), yeast extract (5 g/L), NaCl (10 g/L), PFASs (500 mg/L), and agar (15–20%), pH 7.0 [24].

Methanol (LC-MS grade, CNW), ammonium acetate (LC-MS grade, Fisher Chemical), ultrapure water (18.2 MΩ cm), 17 perfluorinated compounds mixed standard solutions (PFAC-MXB), 9 perfluorinated compounds mixed internal standard solutions (MPFAC) (Wellington Laboratories, Guelph,,Canada). Other chemical reagents were purchased from Tianjin Kemiou Chemical Reagent Co., Ltd (Tianjin, China).

### 2.3. Strains Screening and Identification

#### 2.3.1. Strain Screening

The soil sample (10 g) was inoculated in the enrichment culture media (200 mL) and kept under 160 rpm oscillation at 30 °C. Every 7th day, an enriched liquid (5%) was added to a fresh enrichment media. The PFASs levels were gradually enhanced to 500 mg/L. Then, bacterial liquid (10 mL) was mixed with inorganic salt media (90 mL) augmented with 50 mg/L PFASs. Screening and enriching were conducted with 4 successive transfers. Post-gradient dilution of enriched bacterial liquid (1 mL) at different rates was used to coat the PFAS-containing plates. The bacteria were propagated at 30 °C, and streaking of good growing colonies was performed. Moreover, 15% glycerol was used to preserve the purified strain at −0 °C [25].

#### 2.3.2. Strain Identification

The selected isolates were identified on the basis of traditional physio-biochemical techniques [26]. The identification of 16S rDNA molecular biology included (1) DNA isolation by FastDNA SPIN Kit for Bacteria (Sangon Co., Ltd., Dalian, China) [27]. (2) PCR amplification of 16S rDNA via universal barcoding forward primer 7F/1540R (5′-CAGAGTTTGATCCTGGCTAGGAGGTGATCCAGCCGCA-3′) and reverse primer 27F/1492R (5′-AGTTTGATCMTGGCTCAGGGTTACCTTGTTACGACTT-3′). The parameters were initial denaturation at 95 °C_5 min, denaturation at 94 °C_30 s, annealing at 30 s_57 °C, extension 30 s_72 °C, cycling 30 times, and final extension at 72 °C_10 min. (3) After cloning into the pMD-18T vector, Sangon Co., Ltd. (Shenzhen, China) was used for sequencing. (4) Generation of the phylogenetic tree [27]; the sequence of 16S rRNA gene was compared via GenBank nucleotide database using BLAST on the National Center for Biotechnology Information (NCBI). Sequences with relevant similarities were imported into CLUSTAL W program, aligned, and corrected manually. The phylogenetic tree was generated by the neighbor-joining (NJ) algorithm with MEGA7.0.26 software.

### 2.4. Effects of PFBS Stress on the Growth Curves and the Electron Transport System Activity (ETSA) of Different Tolerant Bacteria

A single colony was picked from the screened plate, inoculated in the LB media, and left at 150 rpm for 72 h at 30 °C. The bacterial suspension was prepared and inoculated into the enrichment culture medium containing PFBS (100 μg/L) with 5% inoculum and cultured at 150 rpm for 7 days at 30 °C, with the pollution-free PFBS as the control (CK). The absorbance (OD) was taken via the UV spectrophotometer at 600 nm to identify the growth of the strains at different times [28].

The ETSA was determined by optimizing Gui’s protocol [29]. Briefly, 0.5 mL of cultured medium was added into a 1.5 mL EP, and 1 mL of 0.2% INT (iodonitrotetrazolium chloride) solution was added to it in the dark for 20 min. Then 50 μL of formaldehyde was used to stop the reaction before spinning (1000 rpm) for 5 min and buoyant removal. The pellet was re-dissolved in 1 mL of 96% methanol, dissolved completely, and spun again at 1000 rpm for 5 min to remove OD interfering bacteria, and its OD was measured at 495 nm with 7 mL of 96% methanol plus 2 mL of 0.2% INT as a blank.

### 2.5. Effects of PFBS Stress on Different Strains’ EPS

#### 2.5.1. EPS Extraction

The stable phase bacterial culture (40 mL) was spun at 2000 r·min^−1^ for 10 min, and the buoyant was discarded, sterile water was added to the original volume, centrifuged again at 2000 rpm for 3 min, and the supernatant was discarded against discard, and sterile water added to the original volume. This was repeated twice to remove impurities in the liquid medium. Then, EPS were extracted by heating the above bacterial suspension at 60 °C in a water bath for 30 min, centrifuging at 8000 rpm for 10 min, and filtering the buoyant through a 0.22 μm membrane to obtain a colorless and transparent EPS solution [22].

#### 2.5.2. PFBS Extraction

A certain amount of culture broth was taken, and 1 ng of PFASs isotopically-labeled standards was added in Ameritech WAX-SPE at a 1 drop/sec flow rate. The WAX solid phase extraction [30] column was mounted before sample loading. Moreover, 4 mL of w = 0.1% ammonia/methanol solution, methanol solution (4 mL), and Milli-Q water (4 mL) were used in the WAX-SPE for activation, respectively. After all samples passed through the WAX-SPE, ρ = 25 mmol/L ammonium acetate buffer (4 mL) was used for washing. The washed WAX-SPE was wrapped in tin foil and centrifuged in a 50 mL tube at 3000 rpm for 2 min for dehydration. The dehydrated WAX-SPE was eluted with methanol solution (2 mL) and w = 0.1% ammonia/methanol solution (2 mL), respectively. The eluate was collected in a 15 mL centrifuge tube and heated in a water bath at 40 °C. The nitrogen rate was adjusted until there was a tiny vortex on the eluate surface, concentrated to 0.5 mL, and then inoculated with 0.5 mL of methanol solution, and the volume was fixed to 1 mL. A 0.22 μm membrane filtered the extract into a 2 mL brown feed bottle and stored at 4 °C for subsequent detection [31].

### 2.6. Analytical Methods

#### 2.6.1. EPS Quantitation

EPS’s protein carbohydrates were measured by a conventional chemical colorimetric test. The protein was quantified by Coomassie brilliant blue using bovine serum albumin as a reference [32]. The carbohydrates were assessed via the anthrone method, keeping glucose as a reference [33].

#### 2.6.2. Chemical Analysis

The PFBS standard was provided by Wellington Laboratories, Inc. It was diluted using water to obtain different concentrations. PFBS was elucidated via ultra-performance liquid chromatography-tandem mass spectrometry (UPLC-MS-MS), attached with a Dionex UltiMate 3000 high-speed pump and Quadrupole Liquid Chromatography system (Thermo Corp., Waltham, USA). The UPLC system included a 2.1 × 100 mm, 3-micrometer Thermo Hypersil GOLD column (Thermo, USA). Additionally, a delay column was also attached after the binary pump to corner the pump system contaminants. The mobile phases were (D) ammonium acetate + water (5 mM) and (A) ammonium acetate + 95% methanol (5 mM). The sample (10 μL) was injected at the flow rate of 0.3 mL/min. The operational gradient started with 20% mobile phase B holding for 1.5 min., then linearly accelerated mobile phase B to 40% in 1.5 min, to 95% in 9 min, and holding in 12 min, then to 80% in 12 min. Then, for 3 min, the column was equilibrated. The total running time was 15 min. The triple quadrupole dMRM acquisition parameters were as follows: SRM negative mode scanning, spray voltage: 3000 V, sheath gas pressure: 50 Arb, auxiliary gas pressure: 10 Arb, atomizer temperature: 350 °C, ion transfer tube temperature: 350 °C, Q1 resolution: 0.7, Q3 resolution: 0.7, CID collision energy: 2.0 mTorr. The target analyte’s information and the details of the PFC’s analytical procedures are presented in Supplementary Materials.

#### 2.6.3. The Fluorescence Spectra Assessment of 3-Dimensional Excitation–Emission Matrix (3D-EEM) and Its Fluorescence Quenching Characterization

The EPS 3D-EEM fluorescence spectra were assessed via a fluorescence spectrophotometer (F-4600, Hitachi, LTD., JAN) by emission spectra of 200 to 550 nm at 5 nm enhanced by altering the excitation wavelength from 200 to 650 nm at 5 nm increments using high sensitivity. Moreover, 12,000 nm/min scanning speed was used. The blank (double distilled water) solution value was subtracted from each sample’s fluorescence value. Results were depicted as emission intensity on a 2D plot, where X-axis = emission wavelength and Y-axis = excitation wavelength. The EEM data were assessed using Cary Eclipse software (Cary WinFLR) [22].

#### 2.6.4. Fourier Transform Infrared (FTIR) Analysis

The extracted EPS were snap frozen (−60 °C, 4 d), and the optimal amount of powder was assessed using NICOLET 380 FTIR system (Thermo Fisher Scientific, Waltham, MA, USA) at 4 cm^−1^ resolution, 400 to 4000 cm^−1^ range, and at transmittance mode [22].

### 2.7. Statistical Analyses

Experiments were replicated thrice, and the values were depicted as mean ± standard deviation. The spectra were baseline corrected in Origin 2015 data analysis and graphing workspace (Origin Lab Corporation, Northampton, MA, USA).

## 3. Results and Discussion

### 3.1. Strain Screening and Identification

After acclimation and enrichment by a PFSA gradient concentration, four strains that utilized PFCs as the only C source were assessed and quantified. Strains NH was cultured in the LB media for 4 days. Their colony was lemon-yellow, round with 1.0–2.5 mm diameter, opaque, and with regular edges. After culturing in inorganic salt media comprising PFBS as a single C source, the colony was yellow, small, round, opaque, and with regular edges. TH strains after LB medium culturing were 1.5–3.5 mm in diameter, round, opaque, khaki-yellow, and with regular edges. After inorganic salt media culturing, the colonies were opaque, small, round, light yellow, and had regular edges. Strain YH colonies after LB medium culturing were 3.0–6.0 mm in diameter, pale yellow in appearance, irregularly round, and translucent. After culturing in the inorganic salt medium, the colonies were large, pale yellow, translucent, with irregular edges. Strain HE colonies after LB medium culturing were 2.0–4.5 mm in diameter, light brown in appearance, irregularly round, and translucent. After culturing in the inorganic salt media with PFBS, the colonies were slightly larger, light brown, translucent, and had irregular edges.

**Figure 1:**
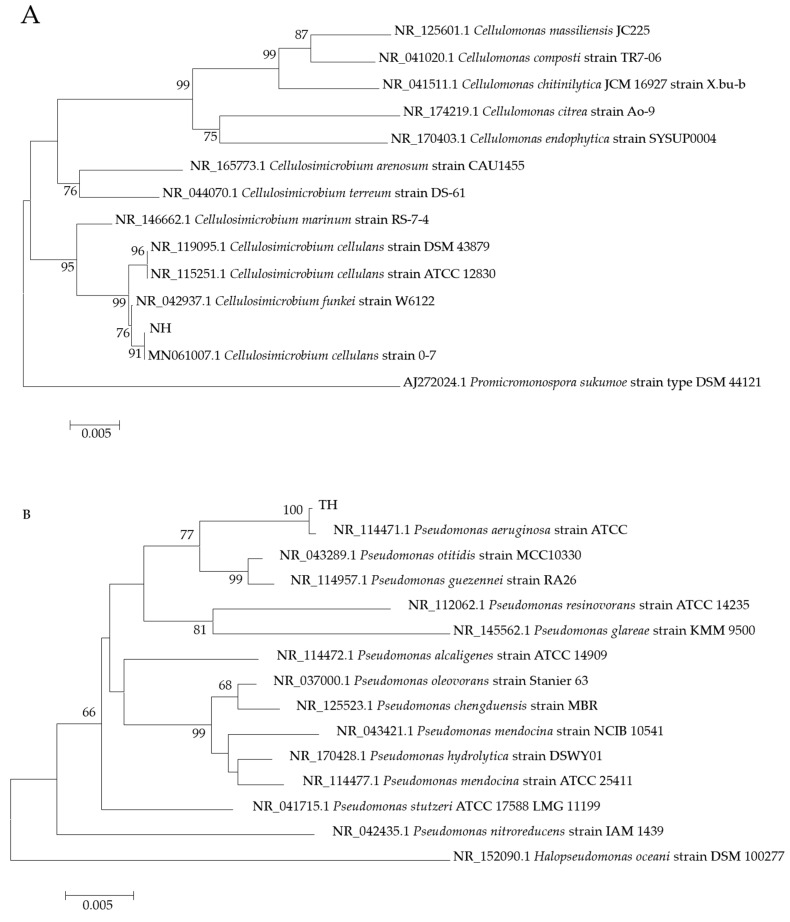

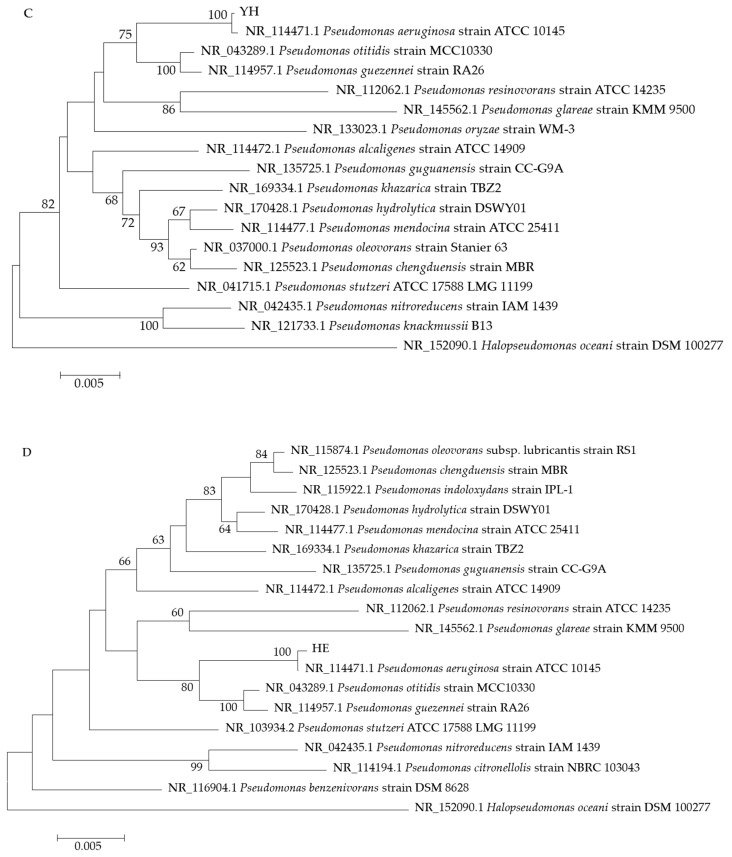
Phylogenetic tree depicting NH (**A**), TH (**B**), YH (**C**), HE (**D**) strain affiliations and related bacterial taxonomy. The tree reveals NJ bootstrap analysis data based on the sequences of 16S rRNA gene. Internal node numbers are bootstrap support values (%). Numbers between brackets are GenBank accession. Halopseudomonas oceani was arbitrarily selected as the outgroup for the phylogenetic tree.

The genomic DNAs of the four strains were extracted, amplified by PCR to obtain 16S rRNA gene fragments, cloned into vectors, and sequenced. Multiple sequence alignments were performed for all four strains. Based on the sequencing data, the homology analysis was performed via the GenBank database, and a phylogenetic tree was built via the MEGA7 (Figure 1). According to the sequence of 16S rRNA genes, NH had high homology with Cellulobacter, and the TH, YH, and HE strains had homology with Pseudomonas. Strain NH clustered in the same clade with *Cellulosimicrobium cellulans* (*C. cellulans*) strain 0–7 (MN061007.1), showing up to 100% identity. According to the colony’s morphology and physio-biochemical factors, NH was preliminarily identified as *C. cellulans*. TH clustered in the same clade with *Pseudomonas aeruginosa* (*P. aeruginosa*) strain ATCC 10145 (NR_114471.1), both showing up to 100% identity. According to the colony’s morphology, physiological, and biochemical features, TH, YH, and HE strains were preliminarily identified as *P. aeruginosa*.

### 3.2. PFBS Stress Effects on the Growth Curves and ETSA of Different Tolerant Bacteria

**Figure 2:**
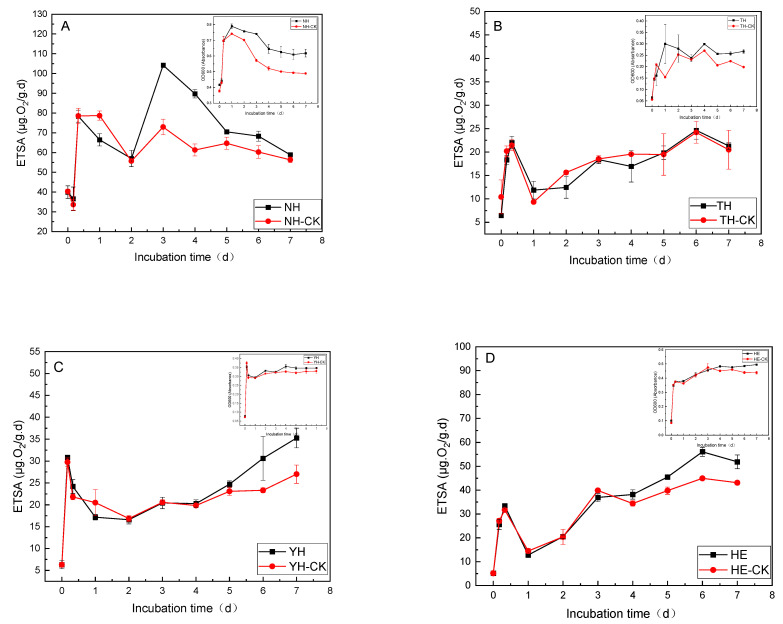
Effects of PFBS stress on the growth curves and ETSA of different tolerant bacteria: (**A**) *C. cellulans* = NH; (**B**) *P. aeruginosa* = YH; (**C**) *P. aeruginosa* Strain TH; (**D**) *P. aeruginosa* = HE.

To investigate the degree of tolerance of four bacterial strains to PFBS, experiments described in Section 2.4 were conducted. The growth and metabolic curves of the tolerant strains (NH, TH, YH, and HE) with PFBS as the main C and energy source were measured using OD_600_ and ETSA as indicators, and the results are depicted in Figure 2A, Figure 2B, Figure 2C and Figure 2D, respectively.

As Figure 2A reflects, PFBS had different degrees of effect on OD_600_ and ETSA of the four tolerant strains. Under the influence of PFBS, the growth of NH reached a maximum value of 0.790 ± 0.014 on day 1 at OD_600_ and 104.187 ± 0.145 μg·O_2_/g·d on day 3 for ETSA, while under no PFBS influence, the growth of the control strain NH-CK reached a maximum value of 0.742 ± 0.000 at OD_600_ on day 1, which was 6% reduced than the growth of the tolerant strain NH on day 1, and ETSA reached 72.96 ± 3.985 μg·O_2_/g·d on day 3, which was 42% lower than the ETSA of the tolerant strain NH on day 3. The NH started to enter the growth stabilization period on day 4, and the OD_600_ after entering the growth stabilization period was always above 0.480, which was markedly higher than the OD_600_ of NH-CK by about 25% and higher than the ETSA of NH-CK by about 30%. Under PFBS influence, the OD_600_ and the ETSA of NH were about 2–3 and 3–4 times greater than the other three strains (TH/YH/HE), respectively. ETSA is the rate of electron transfer along the respiratory chain of microorganisms and is measured based on INT, which is commonly used as an electron acceptor to assess the activity of respiratory dehydrogenase of heterotrophic microorganisms [29]. Since ETSA primarily comprises both substrate and endogenous respiratory metabolic activities, the added contaminant can act as an electron donor to accelerate substrate ETSA release. Organic compounds present in the environment can be toxic to organisms as they accumulate within and damage cell membranes, thereby inhibiting fundamental metabolic processes such as respiration and cell growth [34]. Under PFBS stress, the biochemical activities (OD_600_ and ETSA) of the four tolerant strains exhibited significant changes. Strains with the capacity to remove PFBS demonstrated enhanced growth rates and improved performance in environments with high concentrations of PFBS, thereby achieving effective results [35]. Thus, it can be seen that the NH strain more suitably survives and grows in PFBS because of the higher number of active microorganisms and their metabolism of organic matter under PFBS stimulation.

### 3.3. Effects on the Content of EPS Components and PFBS Removal Rates of Different Strains under the Stress of PFBS

**Figure 3:**
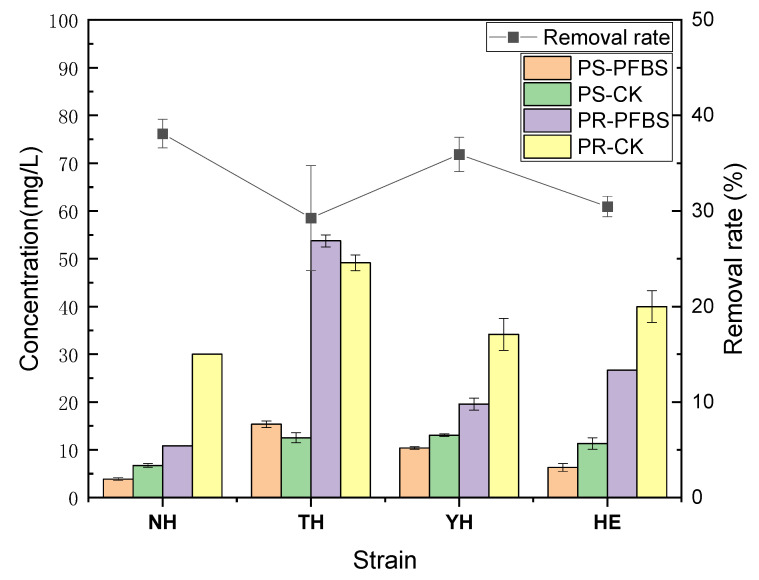
Effects on the content of EPS components and PFBS removal rates of different strains under the stress of PFBS. (PR-CK; protein with the pollution-free PFBS as the control, PR-PFBS; protein with the polluted PFBS, PS-CK; polysaccharide with the pollution-free PFBS as the control, PS-PFBS; polysaccharide with the polluted PFBS).

In this investigation, the strains NH, TH, YH, and HE screened from PFASs contaminated sites were tolerant to high PFBS concentrations and could adsorb a certain amount. In the tolerant strains under the PFBS stress (100 μg/L), the amount of PFBS removal by NH, TH, YH, and HE at day 4 was 38.10%, 29.26%, 35.92%, and 30.48%, respectively (Figure 3). Under PFBS stress, the removal rates of the four tolerant strains exhibited significant differences. Based on the biochemical activities (OD600 and ETSA) of the various strains discussed in the previous section, it can be inferred that higher biochemical activity correlates with improved PFBS removal efficiency among the tolerant strains. Yi et al. [25] isolated PFOA-degrading strain YAB1 from soil near the PFASs producing plant by domestication and enrichment propagation. The PFOA degradation rate was 32.4% after 96 h. Using PFOA as the single C provider, Han et al. [36] identified *Pseudomonas aeruginosa* strain H6 from activated sludge under increased screening pressure by UV and nitrite mutagenesis with ampicillin, the linear alkylbenzene sulfonates (LAS) degradation rate was 44.91% elevated than the original strain. The EPS protein and polysaccharide content was calculated and analyzed to elucidate the specific PFBS-bound organic compounds (mainly protein, polysaccharide, and humic acids). The four tolerant strains significantly affected the protein and polysaccharide content of the major EPS components under PFBS stress, with the NH having a greater effect on the content of major EPS components than the other three strains. Under PFBS stress, the protein content of strains NH, YH, and HE decreased significantly, with reductions of 63.89%, 42.68%, and 33.33%, respectively. In contrast, the protein content of strain TH increased by 9.32%, suggesting that, generally, protein concentration decreases under PFBS stress. Concurrently, the polysaccharide content of strains NH, YH, and HE also experienced significant decreases, with reductions of 42.25%, 20.68%, and 44.21%, respectively. However, strain TH showed an increase of 22.65% in polysaccharide content, indicating that polysaccharide concentration also tends to decrease under PFBS stress. After strain NH was exposed to PFBS stress, the protein and polysaccharide contents in the EPS were significantly reduced. This suggests that the proteins and polysaccharides in the EPS of strain NH are more likely to bind to PFBS, thereby providing additional binding sites that facilitate the removal of PFBS. Zhou et al. [37] found that certain functional groups, such as hydroxyl groups and amides, which are present on activated sludge and other polysaccharides on the bacterial surface, are associated with protein composition. The competitive adsorption of per- and polyfluoroalkyl substances (PFASs) in activated sludge is influenced by the ionic head groups and carbon–fluorine (C–F) chains of these substances. Specifically, PFASs containing sulfonic acid groups and long carbon–fluorine chains are preferentially adsorbed onto sludge. Their synergistic effects, facilitated through hydrophobic interactions and micelle formation, can enhance the overall adsorption capacity of the mixed system. Additionally, Liu et al. [38] identified that extracellular polymeric substances (EPS), the primary component of activated sludge, contain a significant amount of protein, which supports the adsorption and accumulation of perfluorooctane sulfonate (PFOS). The ease of interaction between proteins and PFOS in sludge EPS may be attributed to the presence of active groups, such as hydroxyl and amino groups, within the protein structure. Yan et al. indicate that the main compound binding PFOA to sediments is the EPS protein. Other compounds, such as humic acid, can also form hydrophobic interactions with the carboxyl groups of PFOA, but due to the weak mono-force, their adsorption effect is not as effective as that of proteins [24].

### 3.4. 3D-EEM of EPS of Different Strains Under PFBS Stress

**Figure 4:**
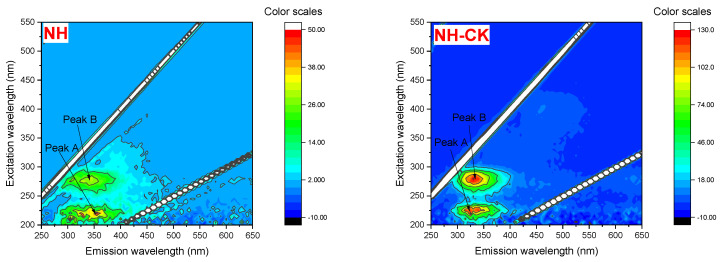

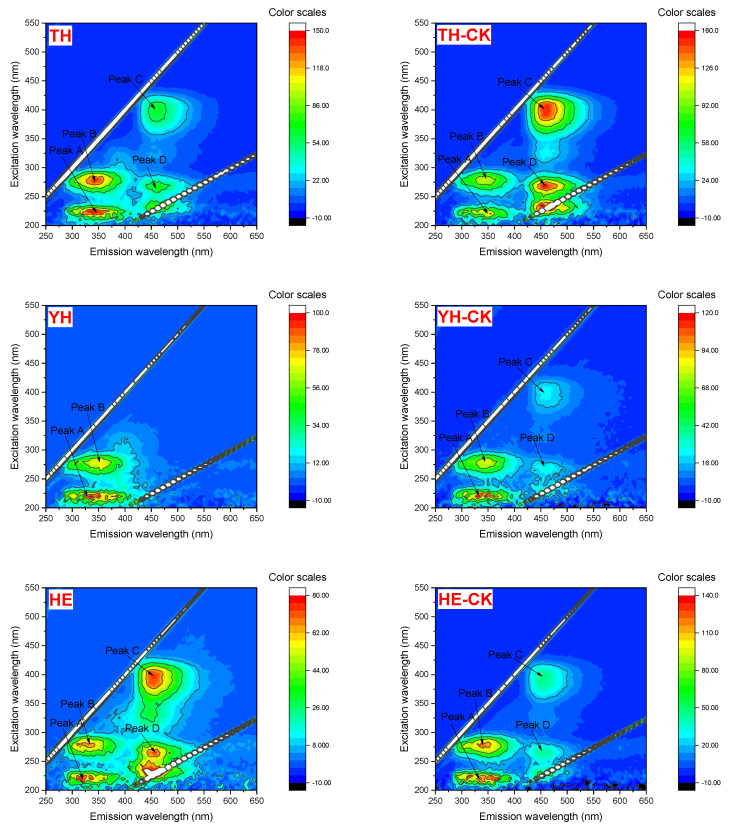
Three-dimensional fluorescence of different strains of EPS under the stress of PFBS.

The 3D-EEM technique was used to determine the direct information on the EPS distribution and composition of each strain. As Figure 4 represents, the fluorescence intensity and EPS distribution of each strain on day 4 under PFBS stress (0 and 100 μg/L). It is observable that the different types of EPS have similar fluorescence peak positions but different intensities.

Hudson et al. [39] and Chen et al. [40] summarized the positions of various dissolved organic matter excitation/emission (Ex/Em) fluorescence peaks in the natural environment; Figure 4 depicts that all four strains had two distinct peaks: A: Ex/Em = 220~250/280~400 nm and B: Ex/Em = 220~300/330~400 nm. The peaks were studied as protein-like peaks, mainly aromatic protein-like substances such as tyrosine (Peak A) and tryptophan protein-like substances (Peak B), proving protein as the main EPS component. Fluorescence Peak C: Ex/Em = 250~400/350~500 nm, located in the region of humic acid-like, and Peak D: Ex/Em = 200~280/330~500 nm, located in the region of fulvic acid, were both found in the YH and HE strains.

The peaks A and B fluorescence intensity of EPS of NH, TH, and HE strains decreased with increasing PFBS concentration (Table 1). In Chapter 3.3, following PFBS stress, the protein content in the EPS of strain TH increased by 9.32%. Consequently, the intensities of fluorescence peaks A and B in the 3D-EEM of strain TH were enhanced. Under the stress of PFBS, the fluorescence intensity of the protein-like peaks of NH was higher than that of the other three tolerant strains, and the changes in A and B peaks intensity were also quite significant, decreasing by 62.5% and 75.8%, respectively. The results indicated that the tryptophan-like and aromatic-like proteins of the NH strain had a strong binding affinity to PFBS, facilitating the adsorption of PFBS by the EPS. Yan et al. [24] found that the adsorption of PFOA by EBPR-EPS was a static quenching process and that the reduction in fluorescence intensity was due to the formation of their complex by hydrophobic binding of the C-F group of PFOA with the tryptophan/tyrosine of EBPR-EPS. In summary, it can be observed that the two fluorescent protein substances, characteristic peaks A and B, are more essential for PFBS adsorption than the humic acid represented by characteristic peak C. Among them, the content of each component of EPS of control tolerant bacteria NH, YH, and HE varied consistently, indicating that the protein dominated the PFBS adsorption process, and the formation and structural stability of tolerant bacteria under PFBS stress depended on the protein fluorescent substances.

### 3.5. FTIR Analysis of EPS of Different Strains Under the Stress of PFBS

**Figure 5:**
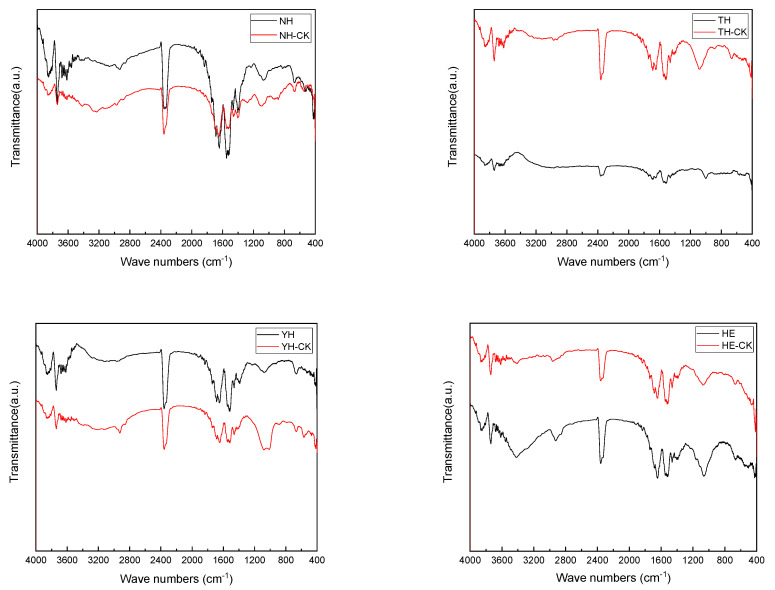
FTIR spectrum of different strains of EPS under the stress of PFBS.

To further illustrate the differences in EPS functional groups of different strains under PFBS stress, an FTIR analysis of each EPS form was used to clarify the functional group’s function and protein structures in the PFBS adsorption by the tolerant strains (Figure 5).

The infrared spectra of PFBS adsorbed by EPS of the tolerant strain (4000–500 cm^−1^) showed that the positions of the characteristic peaks before and after PFBS stress remained unchanged, but the peak intensity enhanced after stress. The peak intensity reflects the relative concentration of the corresponding functional group [41]. The EPS of tolerant strains had a broad absorption band ranging from 3650 to 3200 cm^−1^, formed by -OH stretching vibrations of carboxylic acids and sugars. The intra- and intermolecular hydrogen bonding of carboxylic acids made the peaks broad and were present in various EPS. 2362–2356 cm^−1^ belongs to the interference peaks caused by CO_2_ in air [42]. The typical amide I (C=O stretching vibration) and amide II (superposition of the bending vibration of -NH with one CN stretching vibration) bands appeared at 1689–1641 cm^−1^ and 1513–1546 cm^−1^, respectively. Moreover, 1398 cm^−1^ is the symmetric vibration of C=O in proteins, 1101–1062 cm^−1^ is the C-O-C, and C-O is the stretching vibration of polysaccharides [43]. The presence of phosphorus- or sulfur-containing groups in the fingerprint region of the FTIR spectrum may be a small number of lipids and nucleic acids in EPS. The variation of these groups indicates that -OH groups intolerant strain’s EPS, amides, and polysaccharides are involved in PFBS adsorption. Yan et al. [24] found that all the peak intensities decreased when the PFOA concentration was 1000 ng/L compared to the original EPS spectrum, indicating that the alcohol -OH group and the amide provide active binding sites for the adsorption of PFOA. The decrease in -OH groups (3900–3600 cm^−1^) OD of NH and YH under PFBS stress may be due to their hydrogen bonding with the sulfonic acid groups of PFBS, which can provide electrons to stabilize the energy shell of hydrogen atoms. The decrease in the amide band intensity energy (1689–1641 cm^−1^ and 1513–1546 cm^−1^) may be due to the electrostatic attraction between the protonated PFBS amine and the negatively charged sulfonic acid group. The best removal rate and the greatest protein content variation were observed for the tolerant bacteria NH, further confirming that PFBS could form hydrophobic interactions with the proteins of the tolerant bacteria because of more hydrophobic active sites in the EPS NH.

## 4. Conclusions

In this investigation, the changes in EPS of the four tolerant strains under PFBS stress were compared and investigated to evaluate the role of EPS in PFBS adsorption. Strain NH (*C. cellulans*) showed stronger PFBS removal ability than TH, YH, and HE (*P. aeruginosa*). It was shown that protein and polysaccharide functional groups are essential for binding PFBS to tolerant strains. Specifically, the aromatic component of proteins can form hydrophobic interactions with C-F groups. The amide group binds to the -COOH head of PFBS by electrostatic attraction, and the -OH group of the polysaccharides binds to the sulfonic acid group of PFBS to form hydrogen bonds. EPS proteins can bind with PFBS through electrostatic interaction, thereby reducing cell surface electronegativity and enhancing the joint action between microorganisms.

Furthermore, because of the presence of hydrophobic groups in proteins and polysaccharides, the changes in their content can alter microorganisms’ surface properties and significantly enhance the active binding site, thereby allowing the adsorption of PFBS by the hydrophobic interaction of resistant bacteria. This research provides insight into the role of multiple tolerant strains’ EPS in eliminating PFBS. It contributes to a better understanding of the transformation/removal of PFBS in the environment.

## Figures and Tables

**Table 1:** Different strains of EPS dimensional fluorescence spectroscopy results.

Strains	PFBS (μg·L^−1^)	Peak A		Peak B		Peak C		Peak D	
		Ex/Em (nm)	Int (a.u)	Ex/Em (nm)	Int (a.u)	Ex/Em (nm)	Int (a.u)	Ex/Em (nm)	Int (a.u)
NH	0	220.0/325.0	139.7	280.0/330.0	132.4	——	——	——	——
	100	220.0/335.0	52.4	275.0/330.0	32.06	——	——	——	——
TH	0	225.0/345.0	121.7	280.0/345.0	127.6	405.0/460.0	185.1	270.0/465.0	183.7
	100	225.0/345.0	160.3	280.0/345.0	145.1	400.0/460.0	65.47	265.0/465.0	72.99
YH	0	220.0/345.0	123.3	280.0/345.0	84.63	400.0/460.0	25.11	265.0/460.0	27.33
	100	220.0/335.0	103.6	275.0/350.0	74.39	——	——	——	——
HE	0	225.0/355.0	130.7	280.0/340.0	129.4	400.0/455.0	48.47	265.0/465.0	47.92
	100	220.0/315.0	80.15	280.0/320.0	66.54	395.0/455.0	74.79	265.0/455.0	75.66

## Data Availability

Data are contained within the article. Further inquiries can be directed to the corresponding authors.

## Supplementary Materials

The following supporting information can be downloaded at: https://www.mdpi.com/article/10.3390/toxics12110797/s1, Table S1: Target analytes of 17 PFCs measured in the present study with QA/QC information.
