# Supplementary material for: Composition and Morphological Characteristics of Extracellular Polymeric Substances of Different Tolerant Bacteria Under Perfluorobutanesulfonic Acid (PFBS) Stress
Source: Toxics. 2024 Oct 31;12(11):797. doi: 10.3390/toxics12110797 (PMC11598757; doi:10.3390/toxics12110797)
Supplement: Supplementary file 1 [file toxics-12-00797-s001.zip › toxics-3217030-supplementary.pdf]

Supplemental table

Table S1 Target analytes of 17 PFCs measured in the present study with QA/QC information.

Analyte	Acronym	Internal standard	Precursor (m/z)	Product (m/z)	Limit of Detection (LOD , ng/L)	Limit of Quantitation (LOQ , ng/L)
Perfluoro-n-butanoic acid (C4)	PFBA	$13C_4$ PFBA	212.9	168.946	3.48	11.60
Perfluoro-n-pentanoic acid (C5)	PFPeA	$13C_4$ PFBA	262.869	218.875	3.87	12.89
Perfluoro-n-hexanoic acid (C6)	PFHxA	$13C_2$ PFHxA	312.87	268.817	1.22	4.06
Perfluoro-n-heptanoic acid (C7)	PFHpA	$13C_4$ PFHxA	362.9	318.889	1.32	4.41
Perfluoro-n-octanoic acid (C8)	PFOA	$13C_4$ PFOA	412.87	368.889	10.61	35.36
Perfluoro-n-nonanoic acid (C9)	PFNA	$13C_5$ PFNA	462.868	418.889	1.89	6.31
Perfluoro-n-decanoic acid (C10)	PFDA	$13C_2$ PFDA	512.87	468.817	1.03	3.42
Perfluoroun-n-decanoic acid (C11)	PFUnDA	$13C_2$ PFUnDA	562.85	518.889	0.84	2.81
Perfluoro-n-dodecanoic acid (C12)	PFDoDA	$13C_2$ PFDoDA	612.87	568.889	1.20	3.99
Perfluoro-n-tridecanoic acid (C13)	PFTrDA	$13C_2$ PFDoDA	662.87	618.817	1.15	3.82
Perfluoro-n-tetradecanoic acid (C14)	PFTeDA	$13C_2$ PFDoDA	712.92	668.875	1.06	3.54
Perfluoro-n-hexadecanoic acid (C16)	PFHxDA	$13C_2$ PFDoDA	812.9	768.889	0.63	2.10
Perfluoro-n-octadecanoic acid (C18)	PFODA	$13C_2$ PFDoDA	912.868	868.804	0.54	1.80
Potassium Perfluoro-1-butanesulfonate (C4)	PFBS	$18O_2$ PFHxS	298.87	80.111	1.08	3.61
Sodium Perfluoro-1-hexanesulfonate (C6)	PFHxS	$18O_2$ PFHxS	398.888	99.071	1.18	3.94
Sodium Perfluoro-1-octanesulfonate (C8)	PFOS	$13C_4$ PFOS	498.85	80.097	18.71	62.38
Sodium Perfluoro-1-decanesulfonate (C10)	PFDS	$13C_4$ PFOS	598.85	99	11.25	37.50
